# Roles of post-translational modifications of UHRF1 in cancer

**DOI:** 10.1186/s13072-024-00540-y

**Published:** 2024-05-09

**Authors:** Lili Gu, Yongming Fu, Xiong Li

**Affiliations:** 1 Key Laboratory of Clinical Precision Pharmacy of Guangdong Higher Education Institutes, The First Affiliated Hospital, Guangdong Pharmaceutical University, Guangzhou, 510699 Guangdong China; 2 Key Specialty of Clinical Pharmacy, The First Affiliated Hospital, Guangdong Pharmaceutical University, Guangzhou, 510699 Guangdong China; 3 NMPA Key Laboratory for Technology Research and Evaluation of Pharmacovigilance, Guangdong Pharmaceutical University, Guangzhou, 510006 Guangdong China; 4 School of Basic Medical Sciences, Guangdong Pharmaceutical University, Guangzhou, 510006 Guangdong China

**Keywords:** PTMs, UHRF1, Protein stability, Biological functions, Drug targets

## Abstract

UHRF1 as a member of RING-finger type E3 ubiquitin ligases family, is an epigenetic regulator with five structural domains. It has been involved in the regulation of a series of biological functions, such as DNA replication, DNA methylation, and DNA damage repair. Additionally, aberrant overexpression of UHRF1 has been observed in over ten cancer types, indicating that UHRF1 is a typical oncogene. The overexpression of UHRF1 repressed the transcription of such tumor-suppressor genes as *CDKN2A*, *BRCA1*, and *CDH1* through DNMT1-mediated DNA methylation. In addition to the upstream transcription factors regulating gene transcription, post-translational modifications (PTMs) also contribute to abnormal overexpression of UHRF1 in cancerous tissues. The types of PTM include phosphorylation, acetylation, methylationand ubiquitination, which regulate protein stability, histone methyltransferase activity, intracellular localization and the interaction with binding partners. Recently, several novel PTM types of UHRF1 have been reported, but the detailed mechanisms remain unclear. This comprehensive review summarized the types of UHRF1 PTMs, as well as their biological functions. A deep understanding of these crucial mechanisms of UHRF1 is pivotal for the development of novel UHRF1-targeted anti-cancer therapeutic strategies in the future.

## Introduction

UHRF1 (Ubiquitin-like with PHD and RING Finger domains 1) is a member of the UHRF protein family [1]. UHRF1 is an epigenetic coordinator bridging DNA methylation and histone modifications, and has been involved in the regulation of a series of biological functions [2], such as DNA replication, DNA methylation, and DNA damage repair. Aberrant overexpression of UHRF1 has been reported in more than ten cancer types, indicating that UHRF1 is a typical oncogene, and plays a critical role in the cancer initiation and progression. Initially, murine UHRF1 (also known as NP95) was identified as a nuclear protein associated with cell proliferation [3], and later human UHRF1 (also known as ICBP90) was found to be a transcriptional regulator of topoisomerase II, which is highly expressed in the proliferating cells, especially in the malignant cells [4].UHRF1 protein is composed of five functional domains, including N-terminal ubiquitin-like domain (UBL domain), Tandem Tudor domain (TTD), plant homeodomain (PHD) finger, SET and RING associated (SRA), and C-terminal really interesting new gene (RING) finger domains. The UBL domain recruits E2-Ub and plays a crucial guiding role in specifically transferring Ub from the E2 ubiquitin binding enzyme/ubiquitin complex to histone H3 [5]. The TTD domain links DNA methylation and histone modifications by binding to H3K9me2/3 [6]. Studies have shown that UHRF1 regulates DNA methylation or gene transcription, by interacting with DNMT1, histone deacetylase 1 (HDAC1), histone lysine methyltransferases G9a or Suv39H1 through its SRA or PHD domain [6-8]. The SRA domain is a key structure of UHRF1 protein, which endows UHRF1 with the ability to recognize and bind the hemi-methylated DNA. UHRF1 ubiquitinates histone H3 and the replication factor PAF15, which recruits DNMT1 to the hemi-methylated CpG dinucleotides in the whole genome [9], thereby maintaining DNA methylation patterns and repressing the gene transcription of tumor suppressor genes (TSGs) [10]. The RING domain possesses E3 ubiquitin ligase activity and plays a crucial role in the ubiquitination of H3K23 during S phase. Moreover, the ubiquitin ligase activity of the UHRF1 RING domain is required for DNMT1 binding to the target sites of methylated genes [11]. In addition to its major roles in epigenetics, UHRF1 as a hub protein regulates signal transduction and biological functions by interacting with such non-histone proteins as PML and EG5.

UHRF1 is highly expressed in the proliferating NIH 3T3 cells, while absent in G0 and G1 phases [12], but UHRF1 expression peaks at late G1 and during G2/M phases in human lung fibroblasts [13]. In the terminally differentiated cells, the activation of UHRF1 is induced by adenovirus E1A and substitutes the overexpression of Cyclin E/CDK2 to induce the reentry of S phase of cell cycle [14]. The artificial overexpression of UHRF1 promotes cell proliferation [14], while UHRF1 depletion leads to G1/S cell cycle arrest by elevating the expressions of p53/p21 genes or p73 gene in HCT116 colon cancer cells, and then activating DNA damage response [15, 16]. Depletion of UHRF1 in HCT116 cells induces the activation of DNA damage response and promotes inhibitory phosphorylation of CDK1, thereby resulting in G2/M cell cycle arrest and caspase 8-mediated cell apoptosis [26]. Moreover, in DU145 prostate cancer cells UHRF1 promotes spindle assembly and chromosome congression in mitosis by catalyzing EG5 polyubiquitination [71]. These data indicates that different roles of UHRF1 in cell cycle depends on cancer cell types.

However, aberrant overexpression of UHRF1 causes DNA re-replication, DNA damage, and even genomic instability, which eventually promotes tumorigenesis and cancer progression. Furthermore, aberrant overexpression of UHRF1 altered DNA methylation patterns, thereby suppressing the gene transcription of TSGs and breaking the balance of oncogenes and TSGs, ultimately promoting cancer progression. UHRF1 overexpression has been observed in a panel of cancer types, including lung cancer, breast cancer, gastric cancer, prostate cancer, colorectal cancer, cervical cancer, pancreatic cancer, bladder cancer, endometrial cancer. UHRF1 plays a crucial role in cancer initiation, progression, metastasis and recurrence, and becomes an ideal drug target since UHRF1 knockdown inhibits tumor progression [16]. Therefore, UHRF1 may be used as a diagnostic biomarker [17, 18], as well as a potential target for the development of anti-cancer drugs [19].

Aberrant overexpression of UHRF1 in malignant tissues and cells can be attributed to the dysregulation of gene transcription or post-translational modifications (PTMs). The gene transcription of *UHRF1* is regulated by several proliferation-associated transcription factors. E2F transcription factors, specifically E2F1 and E2F8, activate the gene transcription of *UHRF1* by directly binding to its promoter region [20, 21]. Additionally, Specificity Protein 1 (SP1) or Forkhead Box M1 (FOXM1), also has been reported to promote the gene transcription of *UHRF1* [22]. The types of PTM of UHRF1 include phosphorylation, ubiquitination, acetylation, glycosylation, and others. PTMs are vital in the field of cancer research and contribute to a deeper understanding of cancer biology and the discovery of new diagnostic biomarkers and therapeutic targets.

Different PTMs of UHRF1, individually or in collaboration, confer the diversity of structure and function of UHRF1 protein [23]. Also, the methylation of non-histone proteins, as a type of prevalent PTMs, plays essential regulatory roles in cell metabolism, transcriptional regulation and DNA damage repair [24, 25]. With the aid of illustrations and tables, we comprehensively elucidated the roles of UHRF1 PTMs in cancer progression. We firstly introduced the types of PTMs of UHRF1. Subsequently, we further investigated the interplay and mutual influences of these diverse PTMs, as well as the development of potential of therapeutic strategies targeting the PTMs of UHRF1.

## PTMs of UHRF1 protein

The functional diversity of UHRF1 is closely correlated with the plethora of PTMs such as phosphorylation, ubiquitination, acetylation, and methylation. These modifications play pivotal roles in the regulation of UHRF1 stability, higher order structure, subcellular localization, and the physical interaction with other proteins, and further regulate cell cycle transition, cell proliferation, as well as DNA damage repair [26, 27]. Such PTMs as phosphorylation and ubiquitination are crucial for the maintenance of protein stability and DNA methylation, and the regulation of cell cycle [28, 29].

### Phosphorylation

**Fig. 1:**
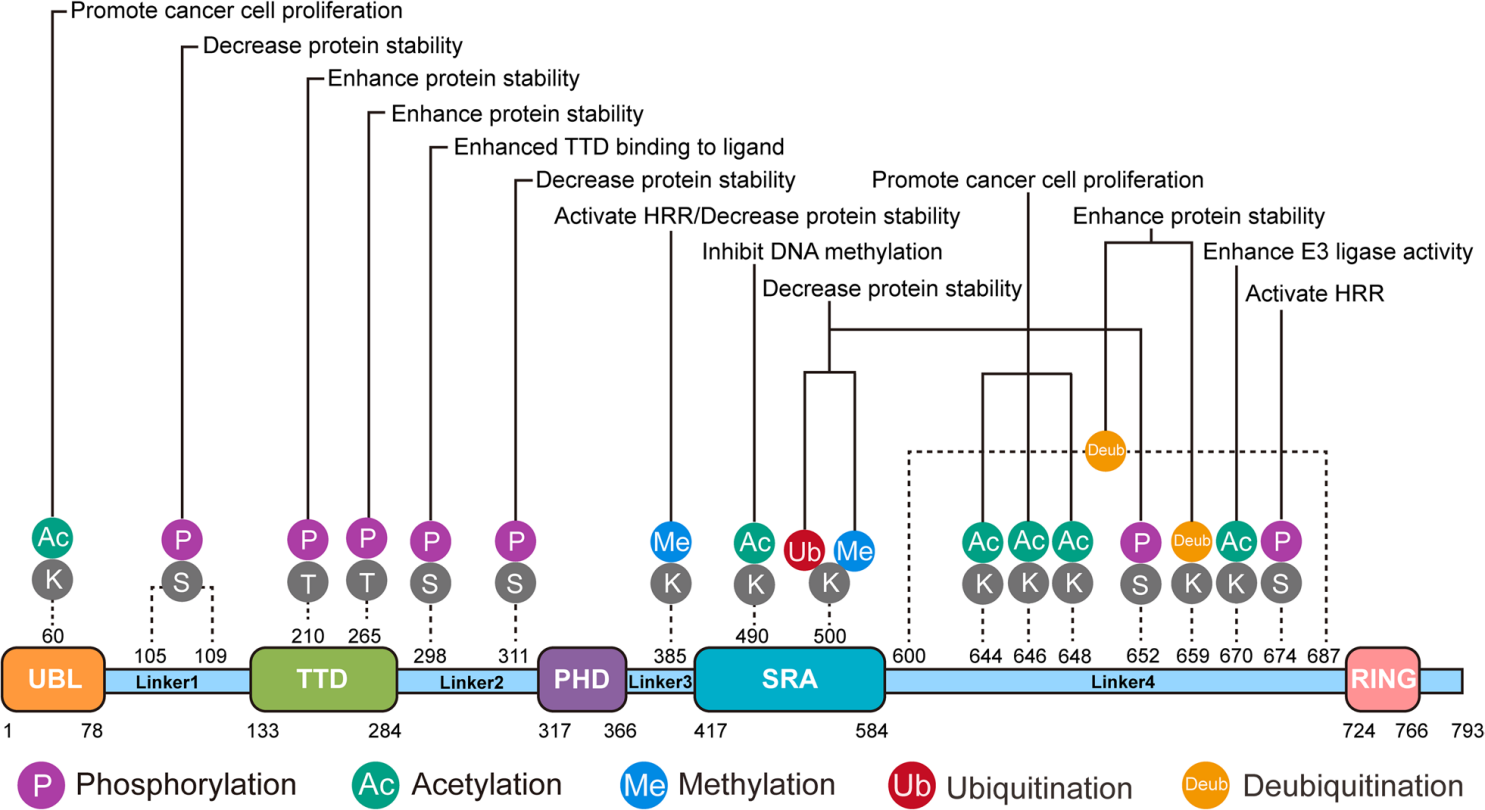
The overview of PTMs sites of UHRF1 (phosphorylation, acetylation, methylation, ubiquitination, deubiquitination), and how these modifications regulate biological functions. Gray indicates the modified proteins (S: Serine, K: Lysine, T: Threonine), and colors represent the types of modifications

Reversible phosphorylation is one type of the most critical PTMs of proteins, typically occurring on serine (S), threonine (T), or tyrosine (Y) residues. Phosphorylation of UHRF1 is primarily responsible for the maintenance of protein stability, subcellular localization and epigenetic regulation. Among the phosphorylation sites of UHRF1, S652 is the first one that has been extensively studied (Fig. 1 and Table 1) [28]. This site is phosphorylated by the M-phase-specific kinase CDK1/cyclin B, thereby preventing UHRF1 from binding to a deubiquitinase Ubiquitin-specific protease 7(USP7), and reducing the protein stability of UHRF1 during M-phase of cell cycle (Fig. 1). Besides, the cells harboring UHRF1-S652A mutation display a decelerated growth, indicating a crucial role of optimal UHRF1 level in the regulation of cell proliferation. In addition, Hao Chen and colleagues [29] reported that in normal or stress conditions such as UV-induced DNA damage, the E3 ubiquitin ligase SCFβ-TrCP induced UHRF1 degradation. In particular, DNA damage triggered the phosphorylation of UHRF1 on Serine 108 (UHRF1-S108) by a casein kinase 1δ (CK1δ), and then promoted SCFβ-TrCP-mediated ubiquitination and eventually caused the protein degradation of UHRF1. Another study reported that the S311 of UHRF1 might be phosphorylated by PIM1 kinase, which then promoted the protein degradation of UHRF1 [30]. Further studies demonstrated that PIM1 kinase destroyed the protein stability of UHRF1 (Fig. 1), thereby reducing the level of DNA methylation, elevating the transcription level of *CDKN2A*, and inducing genomic instability and cellular senescence. Albeit no significant difference compared to wild-type UHRF1, the UHRF1-S311A mutation relieved PIM1-induced senescence. UHRF1 is an inaugural methyl-regulatory factor regulated by PIM1, thereby establishing a connection between PIM1-induced cellular senescence and DNA methylation. These results indicated that downregulation of UHRF1 is a crucial mechanism in PIM1-mediated cellular senescence.

**Table 1:** The confirmed post-translational modifications in human UHRF1

Site on UHRF1 protein	Location in UHRF1 structure	Type of PTM	Team
S652	Linker 4	Phosphorylation	Honghui Ma et al. [28]
S298	Linker 2	Phosphorylation	Satomi Kori et al. [32]
S311	PHD	Phosphorylation	J Yang et al. [30]
S105-109	Linker 1	Phosphorylation	Hao Chen et al. [29]
S674	Linker 4	Phosphorylation	Haoxing Zhang et al. [38]
S265	TTD	Phosphorylation	Yuchong Peng et al. [39]
T210	TTD	Phosphorylation	Yongming Fu et al. [31]
K60, K644, K646, K648	UBL, Linker 4	Acetylation	Ye Joo Hong et al. [43]
K490	SRA	Acetylation	Ja Young Hahm et al. [40]
K670	Linker 4	Acetylation	Linsheng Wang et al. [42]
K385	Linker 3	Methylation	Ja Young Hahm et al. [44]
K385、K500	Linker 3, SRA	Methylation	Huifang Zhang et al. [45]
K500	SRA	Ubiquitylation	Huifang Zhang et al. [45]
/	RING	Ubiquitylation	Abdulkhaleg Ibrahim et al. [51]
600–687	Linker 4	Deubiquitination	Honghui Ma et al. [28]
K659	Linker 4	Deubiquitination	Zhi-Min Zhang et al. [59]

In addition to the regulatory roles in cell cycle, our laboratory has recently reported that AKT1 [31], by inducing the phosphorylation of threonine 210 of UHRF1, maintains the protein stability of UHRF1 (Fig. 1). Further studies have revealed that the phosphorylation of T210 of UHRF1 promotes the interaction of USP7 with UHRF1 (Table 1), while prevents the ubiquitination of UHRF1 by SCFβ-TrCP. AKT1 contributes to the resistance to enzalutamide (abiraterone) in prostate cancer (PCa) by modulating the phosphorylation of UHRF1. The drug resistance may be reversed by AKT inhibitor MK2206 by inducting UHRF1 degradation. These findings provide a potential therapeutic approach for castration-resistant prostate cancer (CRPC) to reverse the resistance of abiraterone.

Furthermore, phosphorylation of UHRF1 regulates its interactions with other partners. Recent studies indicated that the phosphorylation of S298 disrupted the binding of the Tandem Tudor Domain (TTD) of UHRF1 to ligands [32]. During G2/M phases of cell cycle, the elevated phosphorylation level of UHRF1 at S298 significantly promoted its binding to DNA replication sites (Table 1). In addition, UHRF1 interacts LIG1 and H3K9me2/me3 at Lys126 in the heterochromatic regions [33–35], and the phosphate group of S298 dissociates Linker 2 from the peptide binding groove of the TTD to permit the other interactors to access the groove (Fig. 1). Phosphorylation also regulate the subcellular localization of UHRF1. Most nuclear proteins relocalize to the cytoplasm after nuclear envelope breakdown, while UHRF1 remains localized on the chromosomes in M phase [36]. The biological significance of chromosomal localization of UHRF1 is unclear yet, but it has reported that the TTD structure of UHRF1 is not necessary to maintain DNA methylation [65]. Phosphorylation of UHRF1-S298 may promote the chromatin binding of UHRF1 but prevent UHRF1 re-localization to the cytoplasm. In a study using zebrafish as model, UHRF1-S661 was phosphorylated by CDK2/cyclin A, thereby promoting UHRF1 translocalization to cytoplasm from nuclei [37]. Additionally, phosphorylation of UHRF1 regulates DNA damage repair pathways, especially homologous recombination repair (HRR). The CDK2/cyclin A complex directly phosphorylates UHRF1 at S674 (UHRF1-S674) in the S phase of cervical cancer cells (Table 1). UHRF1 phosphorylation is essential for its interaction with BRCA1, a key protein for the activation of DNA HRR (Fig. 1). UHRF1 with the phosphorylated S674 is recruited to double-strand DNA breaks (DSBs) by BRCA1 [38]. Subsequently, UHRF1 mediates K63-linked polyubiquitination of RIF1, and results in its dissociation from 53BP1, thereby facilitating the initiation of HRR. UHRF1-S674A mutant disrupted the association between UHRF1 and BRCA1. However, the UHRF1-S674A mutant does not interferes with its function in regulating DNA methylation, gene expression, and histone H3 monoubiquitination [38].

Moreover, recent studies of our team revealed that PLK1, through the phosphorylation of UHRF1 at S265, facilitated the association between UHRF1 and deubiquitinase USP7. This interaction inhibited the ubiquitination of UHRF1, thereby preserving the protein stability of UHRF1 (Fig. 1). During S phase of cell cycle, PLK1 kinase induced phosphorylation of UHRF1, thereby promoting the binding of UHRF1 to DNMT1. This interaction sustained DNA methylation, suppressed the expression of TSGs, and further preserved cellular vitality [39].

### Acetylation and methylation

The acetylation of UHRF1 is crucial for the regulation of hemi-methylated DNA binding and maintenance of genome-wide DNA methylation. The acetylation of UHRF1 is regulated by P300/CBP-associated factor (PCAF) and HDAC1 [40]. Initially, UHRF1 is acetylated by PCAF at K490, thereby disrupting its binding to the hemi-methylated DNA in the nascent DNA. Conversely, UHRF1 is deacetylated by HDAC1 at K490, which is required for the chromatin association of UHRF1 during S phase. Therefore, UHRF1 acetylation is required for DNA methylation maintenance in colon cancer cells by binding to the hemi-methylated DNA (Fig. 1). Besides, during mouse preimplantation development, HDAC1 maintains genomic methylation by assisting UHRF1 and DNMT1 [41]. A recent study shows the acetylation of the UHRF1 K670 by MOF enhances the activity of the UHRF1 E3 ubiquitin ligase (Fig. 1), and this acetylation is essential for maintaining the normal function of DNA methylation [42]. Then, HDAC1 deacetylates UHRF1, thereby reducing the ubiquitination of histone H3 mediated by UHRF1, leading to the decreased recruitment of DNMT1 and DNA methylation. The acetylation of UHRF1 mediated by MOF/HDAC1 is cell cycle-dependent, and peak in the G1/S phases. Additionally, UHRF1 K667 and K668 can also be acetylated by MOF when K670 is mutated. In addition, it has recently been revealed that UHRF1 is acetylated by TIP60 at the lysine residues of Linker 4 (K644, K646, K648) and UBL domain (K60) [43] (Table 1). Mutation of the acetylation sites by TIP60 elevates the expression of tumor suppressor gene JDP2, and promoted the proliferation of colon cancer cells. Therefore, the inhibition of UHRF1 acetylation may become a potential anti-cancer therapeutic strategy.

Similar to acetylation, methylation is another crucial reversible epigenetic modification occurring on lysine (K) or arginine (R) residues of histone or non-histone proteins. Among the lysine methyltransferases [44], SET domain-containing 7 (SET7) is one of the most extensively studied. UHRF1 methylation may play a role in DNA damage repair (Fig. 1). When DNA damage occurs, UHRF1 is methylated by SET7 at the K385, thereby promoting its binding to DNA damage sites. Reversely, a lysine specific demethylase (LSD1) reduces the levels of UHRF1 methylation. SET7-mediated methylation of UHRF1 at K385 enhances HR repair at the sites of DSB. It is shown to be essential for cell viability against DNA damage.

**Fig. 2:**
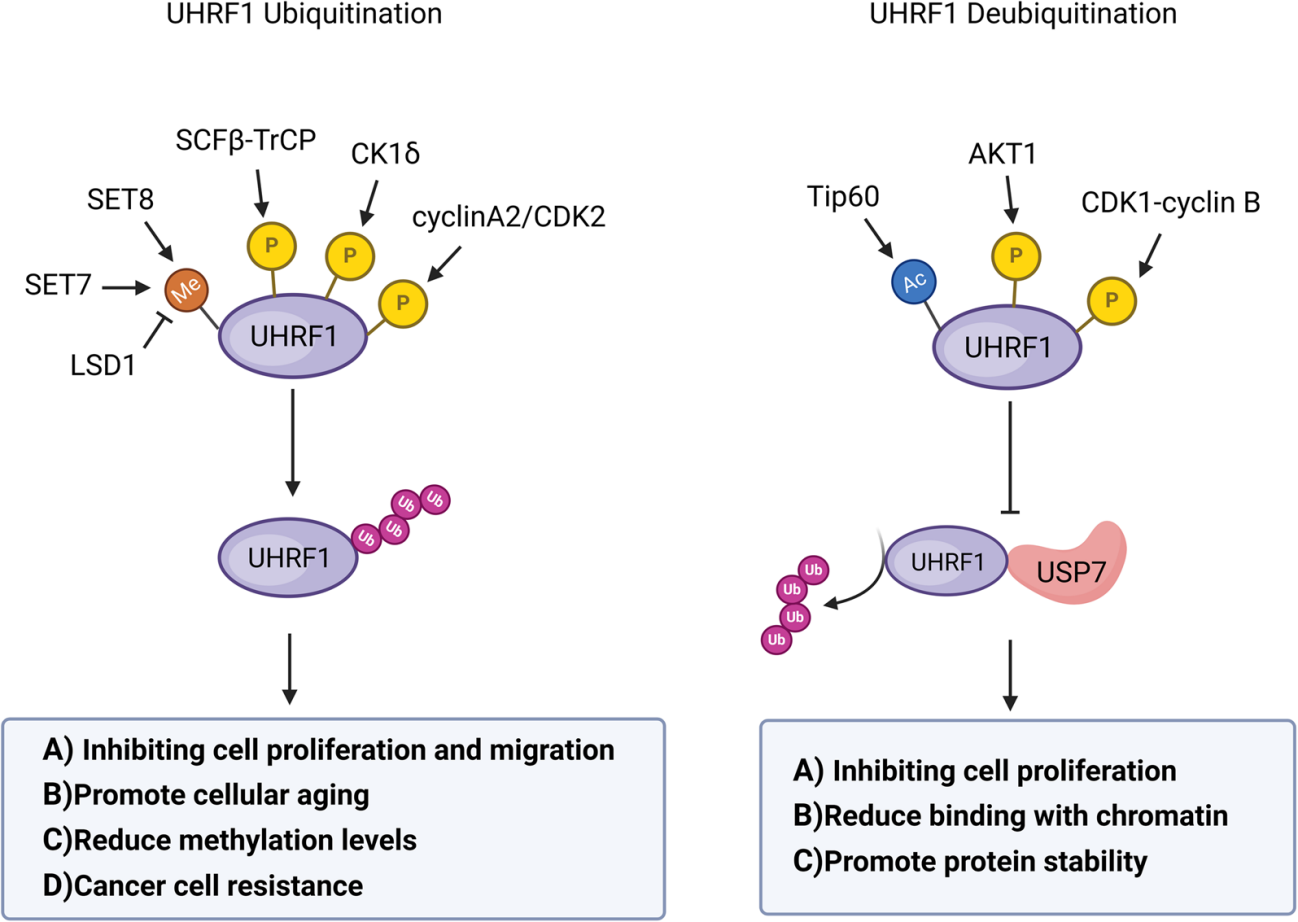
Crosstalk of UHRF1 PTMs. The impact of phosphorylation, methylation, and acetylation on UHRF1 ubiquitination or deubiquitination. These proteases affect the ubiquitination of UHRF1 through UHRF1 PTMs, affecting downstream functions of UHRF1, including inhibiting cell proliferation and migration; Promote cellular aging; Reduce methylation levels; Cancer cell resistance. The deubiquitination of UHRF1 affects its downstream functions, including inhibiting cell proliferation; Reduce binding with chromatin; Promote protein stability. Black arrows indicate the positive effects (cooperative modification crosstalk); Black perpendicular bars indicate the negative effects (antagonistic modification crosstalk)

UHRF1 methylation regulates UHRF1 protein stability. UHRF1 may be methylated by SET8 [45], resulting in the ubiquitination and subsequent protein degradation of UHRF1. Mechanistic studies have revealed that the methylation at UHRF1-K385 or ubiquitination at UHRF1-K500 is essential for SET8-mediated ubiquitination and protein degradation of UHRF1 (Fig. 1 and Fig. 2). Similarly, LSD1 may reverse ubiquitination and protein degradation of UHRF1 by inducing the demethylation of UHRF1. SET8 is a cell cycle-dependent protein methyltransferase that regulates the protein stability of UHRF1 and DNMT1 through the methylation-mediated and ubiquitin-dependent degradation, thereby preventing excessive DNA methylation. SET7/8 inhibitors have already been developed to treat cancer in the preclinical studies, since they exerts anti-tumor efficacy by destroying UHRF1 protein stability [46, 47].

### Ubiquitination and deubiquitination

Ubiquitination is a process covalently linking 76 amino acids ubiquitin (Ub) tags to a target protein. Ub exists in the free form or can be conjugated to a protein as a single *(i.e*., monoubiquitination) or a multiple ubiquitin (*i.e*., polyubiquitination). Ubiquitination is a three-step process catalyzed by ubiquitin-activating enzyme(E1), ubiquitin-conjugating enzyme(E2), and ubiquitin protein ligase(E3) which is responsible for substrate recognition. Ubiquitination regulates protein degradation, subcellular localization of protein, or participates in protein–protein interactions, thereby regulating profound effects on various cellular processes, including signal transduction, cell cycle transition, DNA repair, and immune responses [48].

Ubiquitinations of UHRF1 are typically catalyzed by an enzyme called Beta-Transducin Repeat Containing E3 Ubiquitin Protein Ligase (BTRC). BTRC as an E3 ubiquitin ligase binds to UHRF1 and forms a protein complex, resulting in the ubiquitination of UHRF1 [29, 49]. The ubiquitination of UHRF1 subsequently changes its protein stability and biological functions. A panel of natural compounds or small molecule inhibitors have been developed to target the ubiquitination of UHRF1 [50]. For instance, lenvatinib promotes the protein degradation of DNMT1 and UHRF1 through the ubiquitin–proteasome pathway, thereby promoting protein degradation. However, the precise mechanisms by which lenvatinib induces the ubiquitination of UHRF1 and DNMT1 remains unclear. Thymoquinone (TQ), a natural anticancer compound that induces the polyubiquitination of UHRF1, then triggers the apoptosis of tumor cells [51]. Moreover, UHRF1 may undergo auto-ubiquitination. This process depends on its RING domain [52]. UHRF1 exerts its effects on epigenetic regulation, DNA methylation, and other biological processes [24]. TQ has also been found to induce the auto-ubiquitination of UHRF1 by downregulating USP7. USP7 safeguards UHRF1 from proteasomal degradation by engaging with its SRA domain through the TRAF-like domain. This interaction is crucial for the maintenance of DNA methylation [53]. Additionally, HSP90 inhibitors have been reported to induce UHRF1 ubiquitination-mediated protein degradation, thereby inhibiting cancer cell proliferation [54].

On the other hand, deubiquitination is the process of removing ubiquitin tags from the target proteins and is primarily mediated by deubiquitinating enzymes (DUBs). This process is crucial for the maintenance of protein stability as it prevents ubiquitination-induced protein degradation, or -changed the functions due to the modification of protein interaction with other protein partners [55]. USP7 is a key deubiquitinase regulating their stability and biological activity [56]. For example, MDM2 interacts USP7 to maintain the protein stability [57], and USP7 deubiquitinates FOXO4, leading to its nuclear localization and attenuation of transcriptional activity [58]. The functions of USP7 are multifaceted in the regulation UHRF1. Firstly, the interaction between the UBL (ubiquitin-like) domain of USP7 and the amino acids 600–687 of UHRF1 results in the deubiquitination of UHRF1, which counteracts ubiquitination (Fig. 1) [28]. Additionally, USP7 interacts with UHRF1 via its UBL domains (UBL1-2) and the Linker 4 (K647-678) of UHRF1 [59]. This interaction facilitates USP7-mediated deubiquitination of UHRF1, thereby disrupting the chromatin association of UHRF1. On the other hand, the binding of USP7 to UHRF1 is closely linked to the regulation of cell cycle [28]. During S phase, the interaction between USP7 and UHRF1 causes UHRF1 deubiquitination. As cell cycle enters mitosis, USP7 is dissociated from UHRF1, and UHRF1 is ubiquitinated and subsequently is degraded through the ubiquitin–proteasome pathway. Cumulative evidences suggest that UHRF1 and USP7 interact to form a complex, which in turn influences UHRF1 functions on chromatin remodeling [60], gene expression, and cellular differentiation, ultimately driving cancer development and progression.

## Crosstalk between PTMs of UHRF1

Proteins exert functions through various types of PTMs. When multiple types of PTMs collaboratively regulate a unique protein, called PTM crosstalk [6]. An individual or collaborative PTMs on the same protein may manifest as either synergistic (one PTM promotes another one) or antagonistic crosstalk (one PTM counters another one) [61]. Different PTMs on the residues of UHRF1 determine its protein stability, biological functions, as well as the interaction with other proteins.

The most prevalent cases of UHRF1-PTM crosstalk are predominantly associated with UHRF1 phosphorylation, which influences its ubiquitination (Fig. 2). The deubiquitinase USP7 inhibits the polyubiquitination of UHRF1 by recognizing the phosphorylation at UHRF1-S652. SCFβ-TrCP recognizes the phosphorylations within the amino acids 105–109 of UHRF1, thereby promoting the polyubiquitination of UHRF1 and protein degradation. For example, AKT1-induced UHRF1 phosphorylation (UHRF1-T210) inhibits the ubiquitination of UHRF1. Phosphorylation of AKT1 promotes the USP7-induced deubiquitination of UHRF1, while concurrently inhibiting the SCFβ-TrCP-mediated protein ubiquitination of UHRF1, which leads to the resistance of PCa cells to abiraterone [31]. M phase-specific kinase CDK1/cyclin B induces the phosphorylation of UHRF1 at S652, thereby reducing the interaction between UHRF1 and USP7, reducing the USP7-mediated deubiquitination of UHRF1 and promoting protein degradation, consequently inhibiting cell proliferation [28]. DNA damage induces the phosphorylation of UHRF1 at S108 by CK1δ, thereby promoting SCFβ-TrCP-induced ubiquitination and protein degradation of UHRF1. UHRF1 degradation inhibits the proliferation and migration of cancer cells, while also facilitate the process of senenscence [29]. In addition, the serine-to-glutamic acid mutation at UHRF1-S664 (S664E) significantly reduces the binding of UHRF1647-687 to USP7, thereby promoting the protein degradation of UHRF1. In addition to phosphorylation, UHRF1-K659 is acetylated by TIP60, a modification that interferes with the interaction between UHRF1 and USP7. This disruption leads to the protein instability of UHRF1, and reduces its association with chromatin, beyond the impact of phosphorylation alone [59].

In the crosstalk between phosphorylation and other PTMs, SET-domain protein methyltransferase family modulates both histone and non-histone methylation, and induce chromatin remodeling (Fig. 2). Reversely, UHRF1 regulates gene expression of SET-domain family genes, ultimately promoting the proliferation and metastasis of cancer cells [62, 63]. SET7-induced UHRF1 methylation promotes the polyubiquitination,of proliferating cell nuclear antigen (PCNA), and promotes the protein interaction of PARP1, thereby promoting HRR. Further studies have shown that during S-phase, UHRF1-S661 phosphorylation by cyclinA2/CDK2 is a prerequisite for SET7-induced UHRF1 methylation at the K385 site. LSD1, a typical demethylase, reduced the methylation level of UHRF1, suggesting that UHRF1 methylation controls DNA damage repair pathway [44].

Furthermore, the methylation of UHRF1 are required for its ubiquitination. SET8-mediated methylation of UHRF1 at K385 and K500 also promotes the ubiquitination and the degradation of UHRF1, while LSD1 reverses the ubiquitination-mediated protein degradation of UHRF1 through a demethylation pathway [45]. Currently, many types of UHRF1-PTM crosstalk remain unexplored and insufficiently elucidated in addition to ubiquitination. Further investigations should focus on other forms of PTM crosstalk of UHRF1 protein, such as the crosstalk of methylation, acetylation and phosphorylation.

## UHRF1 mediates non-epigenetic regulation

Epigenetics has always been a highly focused field, playing a crucial role in the regulation of gene expression and cellular function. The achievements in this field are fully demonstrated in the development of "epigenetic drugs" to target epigenetic factors for disease treatment, including cancer. However, epigenetics is only a part of gene expression regulation. In addition to epigenetic factors, non-epigenetic mechanisms also play a crucial role in cell biology, such as cell apoptosis, genomic stability, tumor cell proliferation, and DNA damage repair. It has been reported that UHRF1 overexpression has been observed in various cancer types, and artificial overexpression of UHRF1 promotes tumorigenesis and cancer progression through DNA methylation [64–66]. However, very few non-epigenetic roles of UHRF1 in cancer have been reported.

### UHRF1 regulates the ubiquitination of non-histone proteins

**Fig. 3:**
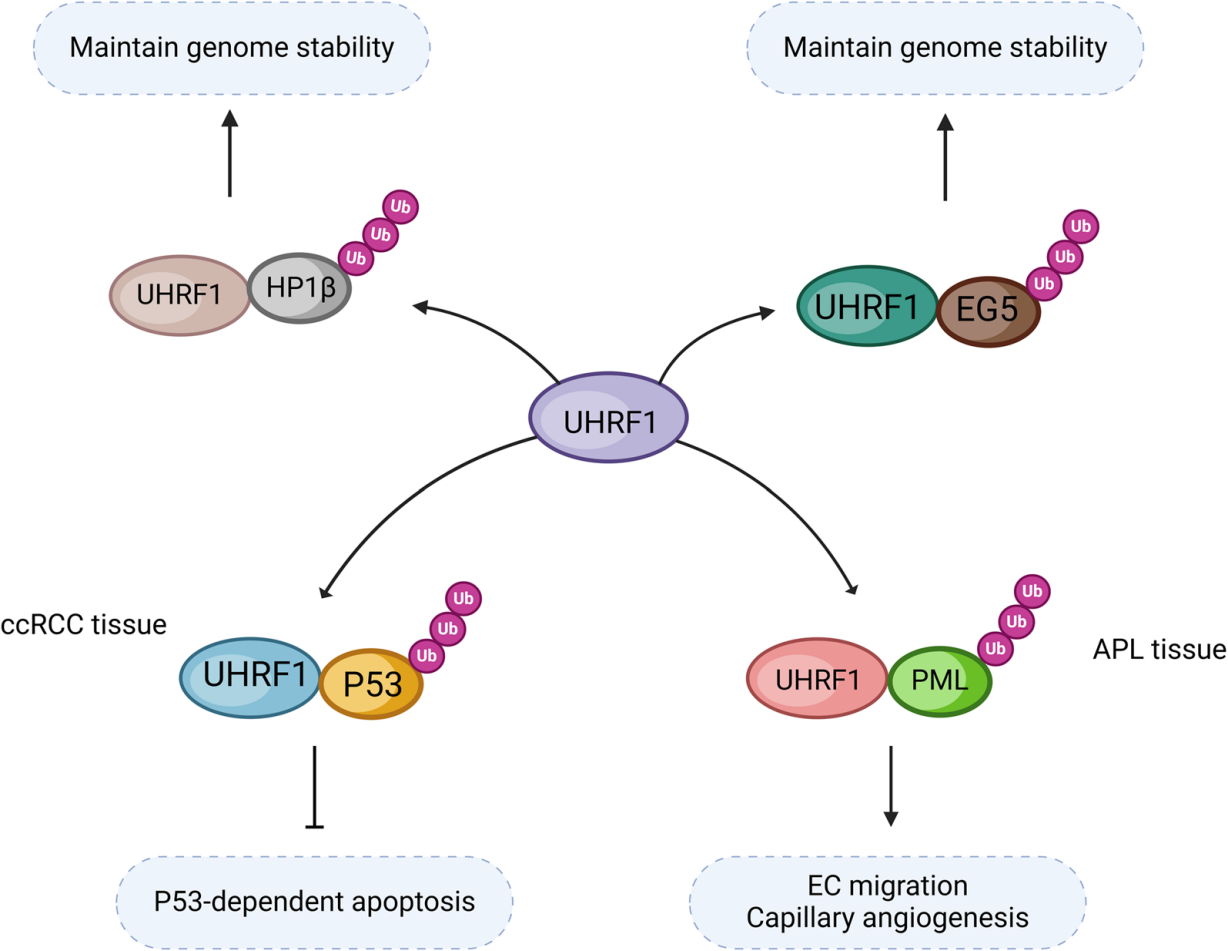
The model illustrates the mechanism by which UHRF1 regulates the ubiquitination of non-histone proteins. UHRF1 induces ubiquitination of its substrate protein, which affects genome stability, cell migration, and apoptosis. HP1β: Heterochromatin Protein 1 beta; EG5: Eg5 kinesin-related motor protein; P53: Tumor Protein 53; PML: Promyelocytic Leukemia Protein

The C-terminal RING domain of UHRF1 possesses an intrinsic E3 ubiquitin ligase activity, indicating UHRF1 is capable of inducing the ubiquitination on other substrates. Ubiquitinations regulate protein stability, subcellular localization, and protein interactions with partner proteins [67, 68]. The ubiquitination of non-histone proteins has contributed to cancer progression. In the tumor tissues of clear cell renal cell carcinoma (ccRCC), UHRF1 interacts with p53, thereby inhibiting the activation of p53 pathway, as well as p53-dependent apoptosis, which is independent of ubiquitination pathway (Fig. 3) [69]. It has recently reported that UHRF1 induces the ubiquitination of HP1β during S-phase, and plays a crucial role in the maintenance of genome stability (Fig. 3), but its mechanism has not been deeply investigated [70]. In a recent publication we revealed an interaction between UHRF1 and the motor protein EG5 during mitosis. UHRF1 triggered the polyubiquitination of EG5 at the K1034 site, preserving the spindle structure and chromosome aggregation essential for the maintenance of genomic stability throughout mitosis (Fig. 3). Moreover, UHRF1-mediated ubiquitination of EG5 promoted its interaction with the spindle assembly factor TPX2, ensuring precise localization to the spindle during metaphase [71].

UHRF1 induces the ubiquitination of several tumor suppressor proteins, thereby is regarded as a potential anti-cancer drug target. PML is a tumor suppressor gene in acute promyelocytic leukemia (APL). The gene fusion of PML with RARA disrupts the functions of PML, and promotes APL development [72]. It has recently been reported that PML interacts with UHRF1, and UHRF1 promotes the migration and capillary formation of endothelial cell (EC) through ubiquitination-mediated PML protein degradation. Therefore, targeting UHRF1 represents a potential therapeutic approach for APL [73].

### UHRF1 regulates signal transduction as a hub protein

As a hub protein, UHRF1 interacts with multiple proteins, thereby assembling multi-protein complexes to regulate signal transduction, gene expression, and other cellular functions.

**Fig. 4:**
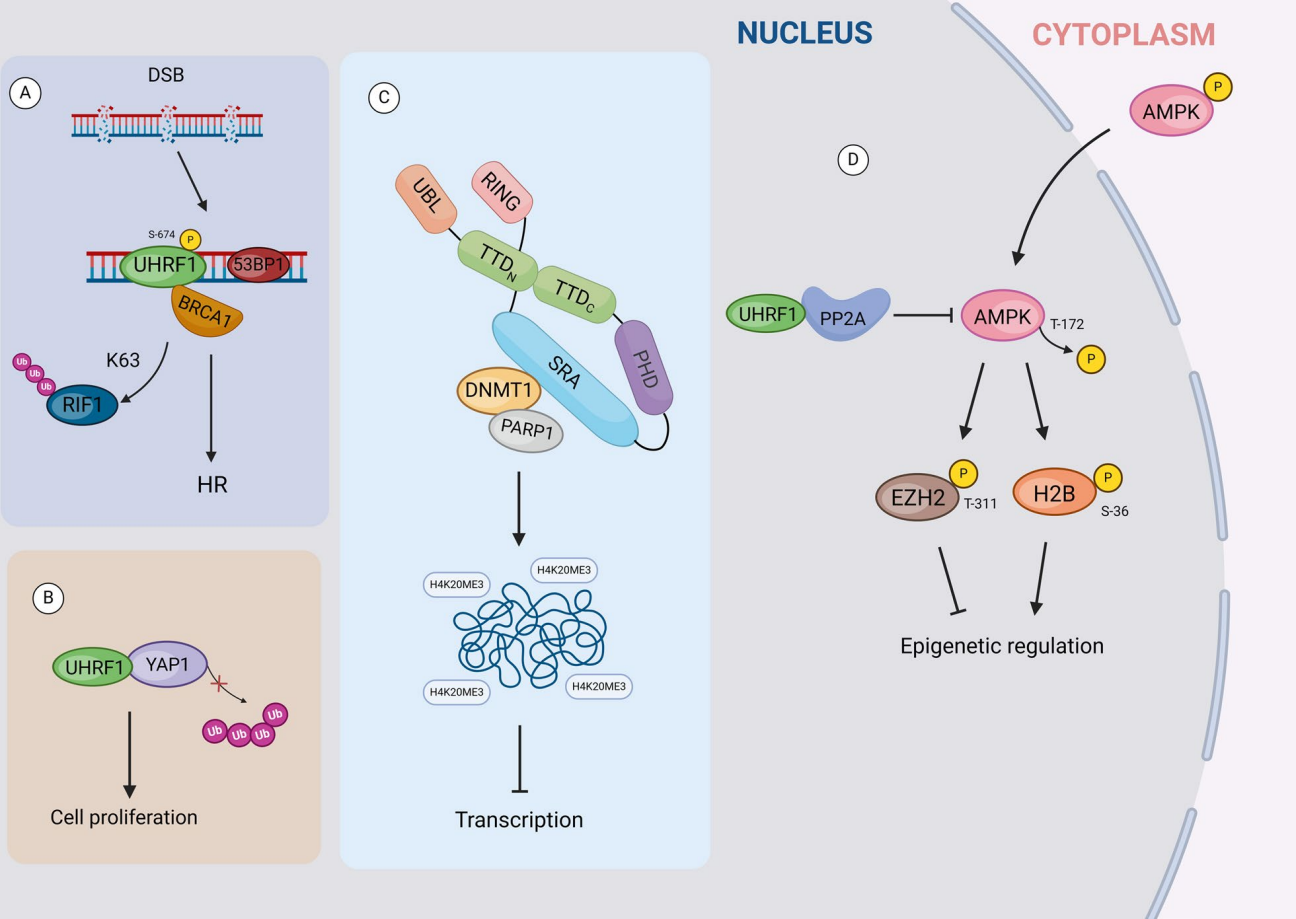
Diagram shows that UHRF1 is a junction protein involved in signal transduction. UHRF1 participates in signal transduction by interacting with different proteins. **A:** The UHFR1-BRCA1 interaction regulates DNA damage response; **B:** The N-terminal binding of YAP1 and UHRF1 promotes cell proliferation; **C:** The PARP1 and UHRF1 SRA domain binding complex inhibits gene transcription activity; **D:** The interaction between PP2A and UHRF1 SRA domain affects the activity of AMPK. YAP1: Yes-associated protein 1; DNMT1: DNA (cytosine-5)-methyltransferase 1; PARP1: Poly (ADP-ribose) Polymerase 1; PP2A: Protein Phosphatase 2A; AMPK: AMP-activated protein kinase; BRCA1: Breast Cancer susceptibility gene 1

It has been reported that UHRF1 controls glucose and lipid metabolism by regulating AMPK activity [74]. Phosphatase PP2A binds UHRF1 inside nuclei, and then UHRF1 induces the dephosphorylation of AMPK at the site T172, thereby inhibiting AMPK activity in both cytoplasm and nuclei. Moreover, UHRF1 suppresses the AMPK-catalyzed phosphorylation of EZH2-T311 and histone H2B-S36. Activated AMPK has been demonstrated to activate stress-responsive gene expression through phosphorylation of histone H2B-S36, controlling the activity of the exonuclease Exo1 to prevent aberrant fork resection during replication stress [75], and inhibiting H3K27 methylation and oncogenic function [76] through phosphorylation of EZH2-T311 (Fig. 4D). They demonstrated in a murine model that UHRF1 controls glucose and lipid metabolism via AMPK. These data suggest that UHRF1 exhibits novel functions in regulating cellular metabolism and coordinating various epigenetic pathways by modulating cytoplasmic and nuclear AMPK activity. Additionally, by interacting with key proteins of DNA damage repair, UHRF1 regulates DNA damage responses. UHRF1 is a critical regulatory factor for the choice of DNA double-strand break (DSB) repair [29]. BRCA1 is an important regulator of DNA damage repair, and UHRF1 interacts with BRCA1 and 53BP1. When DNA double-strand breaks occur, UHRF1 is recruited to the DSB sites during S-phase, and UHRF1-S674 was phosphorylated by CDK2/cyclin A complex, which promotes UHRF1 interaction with the BRCT domain of BRCA1 [38]. Subsequently, UHRF1 mediates K63-linked polyubiquitination of RIF1, and causes its dissociation from 53BP1 and DSBs, thereby promoting the initiation of HRR. Proper repair of chromosomal DSBs is crucial for maintaining genome stability and preventing tumorigenesis (Fig. 4A).

Furthermore, UHRF1 interacts with transcription factors and histone modification enzymes to regulate gene expression. UHRF1 regulates chromatin modifications, which subsequently influences the transcriptional activity of genes. PARP1 is one of protein complex containing UHRF1 and DNMT1 proteins [77]. PARP1 negatively regulates the ubiquitin ligase activity of UHRF1 on DNMT1, introducing PARP1 as an additional regulatory factor for DNMT1 abundance during the S and G2 phases. Then, the PARP1-UHRF1 complex promotes the accumulation of H4K20me3 in heterochromatin, thereby inhibiting abnormal transcription activation (Fig. 4C).

Moreover, UHRF1 has also been involved in the regulation of apoptosis (programmed cell death). UHRF1 induces apoptosis by interacting with apoptosis-related factors. UHRF1 overexpression has been found in various sub-types of lung cancer, and can be used for the precision diagnose of lung cancer [78]. UHRF1 expression levels are significantly elevated in small cell lung cancer (SCLC) and are closely associated with poor prognosis [79]. Mechanistically, UHRF1 binds to YAP1 (the major downstream effector protein of the Hippo signaling pathway) and inhibits the ubiquitination-dependent protein degradation, thereby stabilizing YAP1 protein in tumor cells of SCLC (Fig. 4B). However, the binding sites of UHRF1 and YAP1 have not been dentified yet. UHRF1 promotes cell proliferation and inhibits apoptosis. UHRF1 interacts with WDR79 in Non-Small Cell Lung Cancer (NSCLC), and the knockdown of WDR79 promotes the ubiquitination levels of UHRF1 protein [80]. Therefore, UHRF1 may become a new therapeutic target. Further studies on UHRF1 functions will help improve the prognosis and DDP sensitivity of SCLC patients. In summary, these studies collectively indicate that UHRF1 has been involved in a spectrum of non-epigenetic regulatory processes. Firstly, UHRF1 promotes genome stability through its E3 ubiquitin ligase activity, and inhibits P53-dependent apoptosis, facilitates cell migration and angiogenesis through promoting PML ubiquitination, and enhances DNA damage repair. Secondly, UHRF1 serves as a scaffold protein to facilitate glucose and lipid metabolism, as well as transcriptional gene activation. Despite some initial findings, the precise mechanisms and clinical significance need further investigation and validation, whch will provide a theoretical basis for the personalized molecule-targeted therapies.

## Conclusions and future directions

Until now more than 726 or 431 publications have been posted in the Pubmed database when “UHRF1” or “UHRF1 and Cancer” was used as the subject word of search. UHRF1 as a prominent molecule in cell biology and epigenetics, plays a crucial role in the maintenance of DNA methylation and chromatin modifications, especially in the development of malignancy. The expression levels and biological functions of UHRF1 are regulated by a series of PTMs in addition to the transcription regulation. Previous studies have demonstrated, the protein stability and biological functions of UHRF1 are regulated by ubiquitination. In addition, phosphorylation regulates the interactions of UHRF1 with other proteins in addition to cellular signaling pathways. Acetylation of UHRF1 modulates chromatin remodeling, as well as the crosstalk of different PTMs of UHRF1. Methylation influences the interactions of UHRF1 with other proteins and the subcellular localization. However, it is worthy of further studying the functional relevance of UHRF1 PTMs in cancer.

Firstly, it is necessary to identify novel types and sites of PTM to understand how they regulate biological functions through the interactive partners. Based upon the available data, future investigations should focus on more PTMs in the RING domain, the methylation in the PHD domain, and acetylation in the TTD domain. The key regulators of PTMs including methylation and acetylation have been reported to bind the individual domains of UHRF1, such as SET7 and SET8 in the PHD domain, and PCAF and p300 in the TTD domain. It is worthy of further investigation to clarify the structural interaction and functional association between UHRF1 and these regulators through individual domains. Other types of PTMs of UHRF1, such as glycosylation, SUMOylation, as well as lipidation, have not comprehensively revealed yet. For instance, glycosylation may occur at the residues of lysine, serine and threonine residues, which regulate the subcellular localization, protein stability, and the interactions with other proteins. Additionally, SUMOylation may also occur on the residues of lysine of UHRF1, and regulates DNA methylation and chromatin modifications, as well as the interactions with other proteins. Lipidation may occur on the residues of lysine of UHRF1, and regulate chromatin remodeling and gene expression, particularly the interactions with the nuclear membrane or chromatin proteins.

Secondly, the crosstalk of different PTMs of UHRF1 makes the regulatory mechanisms more complicated, and is worthy of further investigation. The PTMs of UHRF1 play critical roles in the regulation of cellular functions, and different types of modifications may intricately intertwine to collectively regulate the biological properties of UHRF1. When UHRF1 is modified by a single PTM type, lysine acetylation is associated with DNA methylation. However, in the most cases, serine phosphorylations promotes the chromatin binding and protein stability of UHRF1. Serine residues may be mono-methylated, asymmetrically or symmetrically dimethylated [81, 82]. The methylation of lysine and arginine may occur in UHRF1 protein itself, and influences biological functions.

Thirdly, further studies are required to reveal the roles of UHRF1 PTMs in cancer initiation and progression, and to unveil their associations with crucial biological processes such as cell proliferation, metastasis, and therapeutic resistance. The studies above illustrated that the PTMs of UHRF1 closely bound to a variety of molecule partners, including DNMTs, histone-modifying enzymes, tumor suppressor proteins (such as p53), and cell cycle regulatory proteins.

Lastly, it is worthy of noting that the most experiments studying UHRF1 PTMs have been conducted in the in vitro system. Therefore, the in vivo experiments are required to further validate these findings and ensure their effectiveness in the clinical applications for cancer treatment.

In conclusion, the studies of UHRF1 PTMs, including the identification of associated modifying enzymes and modification residues, as well as the interactions with other proteins, will benefit for deeper understanding of the regulatory mechanisms of UHRF1, in the physiological or pathological conditions, and for the development of novel UHRF1-targeted anti-cancer therapeutic strategies.

## Data Availability

Data availability is not applicable to this article as no new data were created or analyzed in this study.

## Abbreviations

BRCA1: Breast Cancer susceptibility gene 1
CDH4: Cadherin 4
HDAC1: Histone deacetylase 1
MOF: MYST histone acetyltransferase
H3K23: Histone H3 Lysine 23
PAF15: Platelet-Activating Factor Acetylhydrolase 15
TSGs: Tumor suppressor genes
E1A: Early region 1A
SP1: Specificity Protein 1
FOXM1: Forkhead Box M1
USP7: Ubiquitin Specific Peptidase 7
SCFβ-TrCP: Skp1-Cullin1-F-box protein β-Transducin Repeat-Containing Protein
CDKN2A: Cyclin-Dependent Kinase Inhibitor 2A
CK1δ: Phosphotase casein kinase 1δ
PIM1: Proviral Integration site for Moloney Murine leukemia virus 1
AKT1: AKT serine/threonine kinase 1
LIG1: DNA ligase 1
HRR: Homologous recombination repair
DSBs: Double-strand DNA breaks
PLK1: Polo-like kinase 1
TSGs: Tumor suppressor genes
PCAF: P300/CBP-associated factor
JDP2: Jun dimerization protein 2
SET7: SET domain-containing 7
LSD1: Lysine specific demethylase
SET8: SET domain-containing 8
BTRC: Beta-Transducin Repeat Containing E3 Ubiquitin Protein Ligase
DNMT1: DNA (cytosine-5)-methyltransferase 1
TQ: Thymoquinone
DUBs: Deubiquitinating enzymes
MDM2: Mouse Double Minute 2
FOXO4: Forkhead Box O4
PCNA: Proliferating Cell Nuclear Antigen
ccRCC: Clear Cell Renal Cell Carcinoma
HP1β: Heterochromatin Protein 1 beta
EG5: Eg5 kinesin-related motor protein
TPX2: Targeting Protein for Xenopus Kinesin-like Protein 2
APL: Acute promyelocytic leukemia
RARA: Retinoic Acid Receptor Alpha
PML: Promyelocytic Leukemia Protein
EC: Endothelial cell
PP2A: Protein Phosphatase 2A
PARP1: Poly (ADP-ribose) Polymerase 1
SCLC: Small cell lung cancer
YAP1: Yes-associated protein 1
NSCLC: Non-Small Cell Lung Cancer
WDR79: WD Repeat Domain 79
